# Cardiac Arrest Due to Pulmonary Embolism After Posterior Spinal Fusion in a Patient With Acute Paraplegia Caused by a Metastatic Spinal Tumor Associated With Congenital Antithrombin III Deficiency

**DOI:** 10.7759/cureus.22618

**Published:** 2022-02-26

**Authors:** Toru Funayama, Toshinori Tsukanishi, Hiroshi Noguchi, Masaki Tatsumura, Masashi Yamazaki

**Affiliations:** 1 Orthopaedic Surgery, Faculty of Medicine, University of Tsukuba, Tsukuba, JPN; 2 Orthopaedic Surgery, Tokyo Medical University Ibaraki Medical Center, Ami, JPN; 3 Orthopaedic Surgery and Sports Medicine, Tsukuba University Hospital Mito Clinical Education and Training Center, Mito Kyodo General Hospital, Mito, JPN

**Keywords:** antithrombin iii deficiency, metastatic spinal tumor, paraplegia, posterior spinal fusion, pulmonary embolism

## Abstract

Congenital antithrombin (AT) III deficiency has a high incidence of deep vein thrombosis and pulmonary embolism due to reduced anticoagulation. In this study, we report a case of a patient who experienced cardiac arrest due to pulmonary embolism after emergency posterior spinal fusion for acute paraplesia due to a metastatic spinal tumor associated with AT III deficiency. A 49-year-old man with a history of familial AT III deficiency visited our hospital due to difficulty in walking caused by a progressive paralysis of the lower limbs. Clinical examination revealed multiple bone metastases due to prostate cancer and spinal cord compression caused by a pathological fracture of the T1 vertebral body. He had low AT III activity levels and high D-dimer levels. The following day, he underwent posterior spinal decompression and fusion. However, he had pulmonary embolism with cardiac arrest three days after surgery. He recovered without sequelae after emergency thrombectomy following resuscitation. Patients with AT III deficiency who cannot walk due to a metastatic spinal tumor inevitably develop deep vein thrombosis and pulmonary embolism. To avoid lethal pulmonary embolism, preventing deep vein thrombosis should be prioritized before surgery, even in the presence of acute progressive paraplegia.

## Introduction

Congenital antithrombin (AT) III deficiency is an autosomal dominant genetic disease that was first reported by Egeberg in 1965 [1,2]. Patients with congenital AT III deficiency have lower AT III activity and a high prevalence of deep vein thrombosis (DVT) and pulmonary embolism (PE) due to reduced anticoagulation caused by the deficiency of anticoagulation factors involved in the coagulation control system [3]. The prevalence of AT III deficiency is 0.02% to 0.2%, [4,5] and it is known to increase in onset after surgery, injury, delivery, and oral contraceptives. Perioperative management focusing on the prevention of thrombosis due to hypercoagulability is mandatory during surgery for patients with AT III deficiency [6]. However, in some cases, spine surgeons hesitate to administer perioperative anticoagulant therapy because of worrying about postoperative complications, such as epidural hematoma, which is requiring emergency surgery.

In this study, we report a case of a patient who experienced cardiac arrest due to PE after emergency posterior spinal fusion for acute paraplesia due to a metastatic spinal tumor associated with AT III deficiency. There is no existing English literature on patients with AT III deficiency who developed PE after spinal surgery. In addition, perioperative management methods for patients with AT III deficiency are lacking. Therefore, we present our case, along with a review of relevant literature.

## Case presentation

A 49-year-old man was diagnosed with AT III deficiency 13 years ago that was left untreated. Two months before the visit, he experienced discomfort in the posterior cervical region; thus, he visited a local clinic. However, no abnormalities were found. At two weeks before the visit, he experienced weakness of lower limbs. He was referred to our hospital due to difficulty in walking for five days caused by sudden progressive paraplesia.

**Figure 1:**
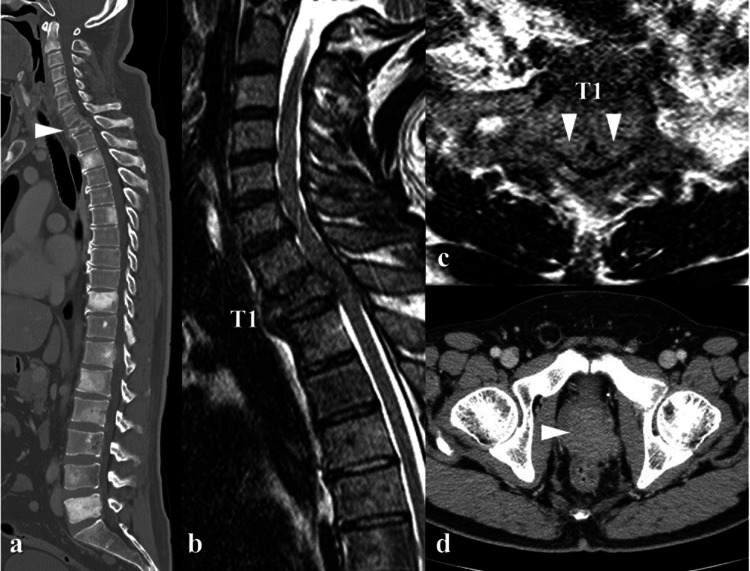
Preoperative images. (a) Whole spinal CT showing multiple osteoblastic lesions and pathologically collapsed T1 vertebral body (arrowhead). (b, c) T2-weighted MRI images showing severe spinal cord compression due to an epidural lesion (arrowheads). (d) CT showing an enlarged prostate (arrowhead).

At the first visit, he was unable to walk. The deep tendon reflexes of the lower limbs (both the patellar tendon and the Achilles tendon) were increased. The right lower limb muscle strength was markedly reduced. The results of the manual muscle test were as follows: hip joint flexion, 2; knee extension, 2; ankle dorsiflexion, 1; dorsiflexion of the hallux, 1; ankle plantar flexion, 1; and left lower limb, approximately 3-4. He had a reduced sensation in the area ranging from the xiphoid process of the sternum to the entire lower limbs and the perineum. He had poor bladder function and dysuria from when he noticed difficulty of walking. Computed tomography (CT) after emergency admission showed multiple osteoblastic metastases in the spine and pathological fracture in T1 vertebral body (Figure 1a). T2-weighted magnetic resonance imaging (MRI) of the T1 vertebral body showed severe spinal cord compression due to epidural metastatic lesions in the spinal canal (Figures 1b, 1c). In addition, CT showed an enlarged prostate (Figure 1d).

**Figure 2:**
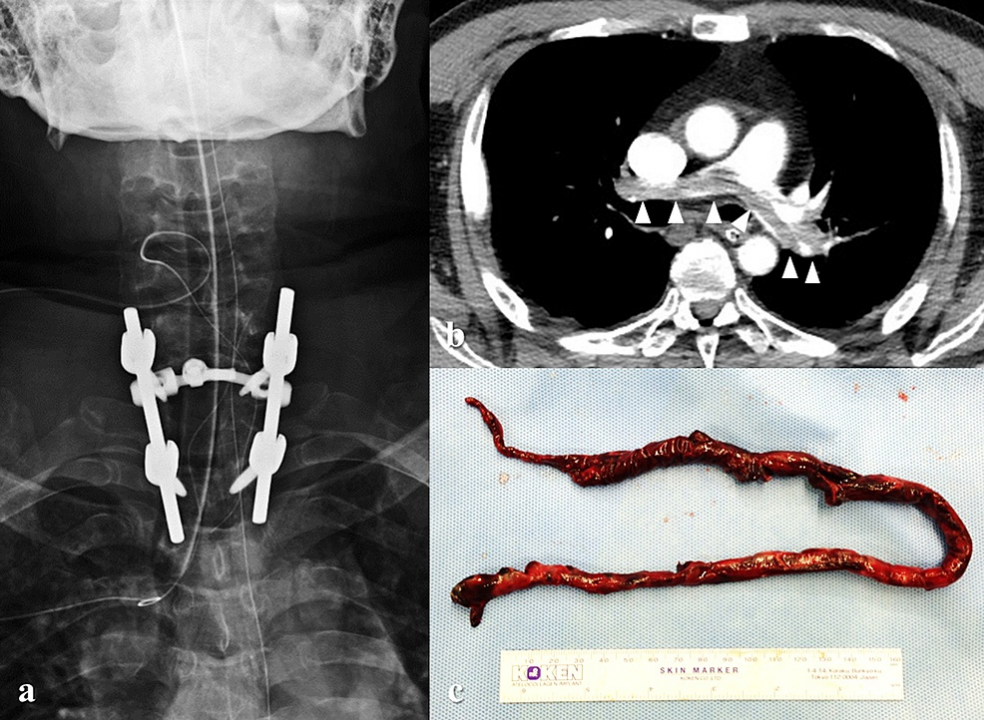
Postoperative images. (a) X-ray taken immediately after spinal surgery. C7-T2 posterior fusion and C7 and T1 laminectomies were performed. (b) Contrast-enhanced CT at three days after surgery. Extensive defect of contrast medium in the area ranging from the main pulmonary artery to the pulmonary artery bifurcation extending into both trunks (arrowheads). (c) The main pulmonary artery was incised to remove a giant thrombus (39 cm).

Blood test showed abnormally high prostate-specific antigen (1,984 ng/mL). AT III activity was reduced (51.7%). In addition, D-dimer increased to 8.5 μg/mL. Therefore, he was diagnosed with acute lower limb paresis (Frankel classification, grade C) due to metastatic spinal tumor of prostate cancer. On the day following admission, C7-T2 posterior fusion with laminectomies of C7 and T1 (Figure 2a), and prostate biopsy were performed. The epidural lesion was partially dissected for a pathological examination. The duration of surgery was 5 hours and 9 minutes, and the bleeding was 290 g. Regarding AT III deficiency, after consulting with a cardiologist in our hospital before surgery, anticoagulant therapy was planned to be initiated once the postoperative epidural bleeding stopped.

D-dimer levels increased to 38 μg/mL on the day after surgery. Subsequent ultrasonography of the lower limb veins showed an old thrombus in both popliteal veins. Therefore, the intermittent pneumatic compression device put on the lower limbs at the start of surgery was removed. At three days after surgery, he experienced acute dyspnea and a convulsive seizure followed by cardiac arrest. Resuscitation was immediately started. Eleven minutes later, ventricular tachycardia was noted. Therefore, electrical cardioversion was performed. On resumption of cardiac rhythm, contrast-enhanced CT showed extensive defect of contrast medium in the area ranging from the main pulmonary artery to the pulmonary artery bifurcation extending into both trunks (Figure 2b). Therefore, he was diagnosed with lethal PE. Despite administering high-dose catecholamines, hemodynamics still did not stabilize. Thus, percutaneous cardiopulmonary support was initiated to stabilize the circulatory dynamics, and emergency cardiac surgery was performed with starting AT III replacement therapy and heparinization. Incision of the main pulmonary artery was made to remove a giant thrombus (39 cm, Figure 2c).

**Figure 3:**
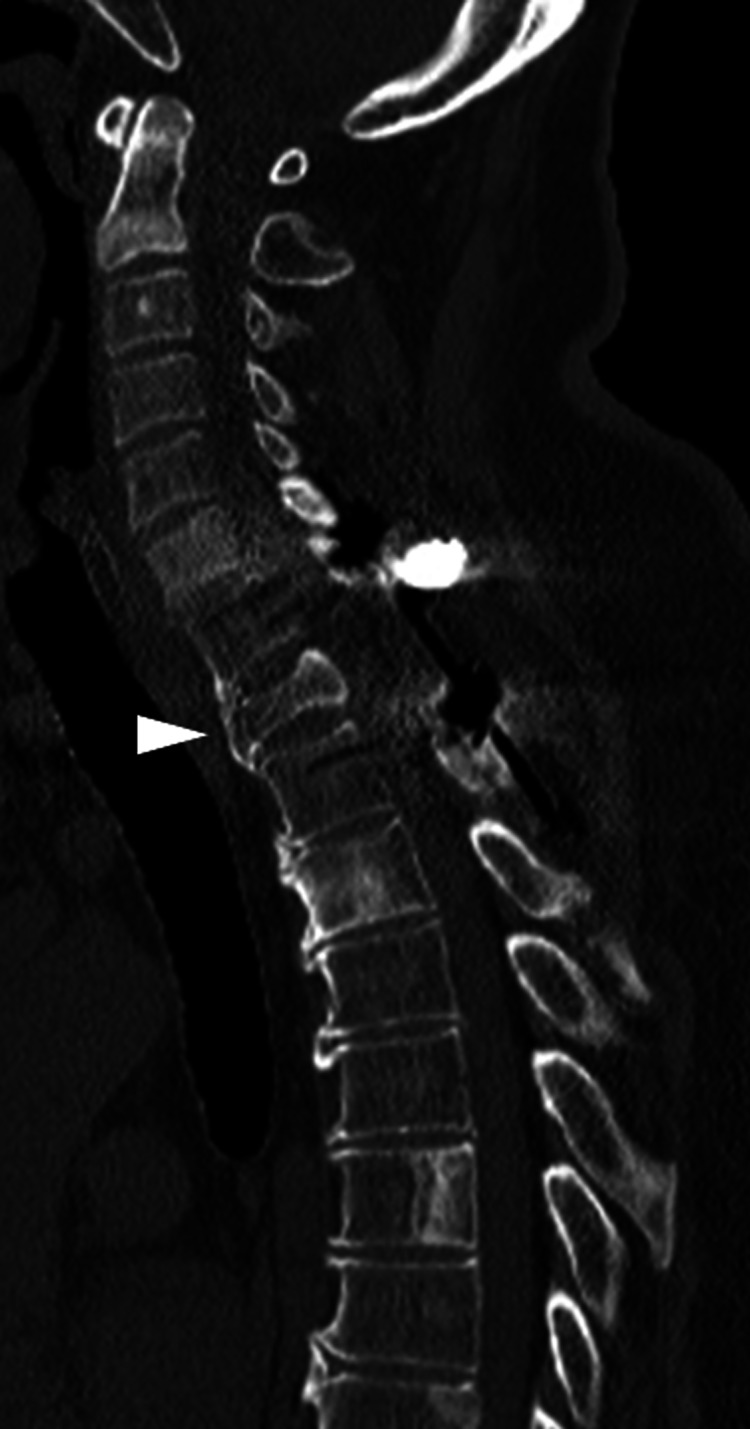
CT images at two years and six months after surgery. Bone union was seen between the T1 and C7 vertebral body with bony bridging (arrowhead).

He underwent therapeutic anti-hyperthermia for brain protection and was withdrawn from the ventilator five days after surgery. Eight days later, a permanent inferior vena cava filter was inserted, and warfarin administration was started. Because paralysis of the lower limbs improved and he was able to walk within parallel bars at five weeks after surgery, he was transferred to a rehabilitation facility. Hormone therapy was initiated for the treatment of prostate cancer. At the final observation at two years and six months after surgery, he was ambulatory without assistance. CT showed bone union between the T1 and C7 vertebral body with bony bridging (Figure 3).

## Discussion

In the Guidelines for the Diagnosis, Treatment and Prevention of Pulmonary Thromboembolism and Deep Vein Thrombosis [7], congenital AT deficiency is classified as the highest risk, requiring strict perioperative control and prevention of thrombosis. In addition, patients with low preoperative AT activity require perioperative administration of AT products to maintain the AT activity at 80%-100% [8,9].

The incidence of perioperative DVT and PE after all spinal surgeries is 0.29% and 0.24%, respectively [10]. However, a high prevalence of DVT at screening has been reported in patients undergoing surgery for metastatic spinal tumors (9.48%), particularly those who were unable to walk (24.4%) [11].

The patient in this study had a history of AT III deficiency. He had low AT activity levels upon admission (51.7%). In addition, he had high D-dimer levels of 8.5 μg/mL, well above the preoperative cut-off value for the risk of thrombosis after spinal surgery (6.5 μg/mL) [12]. Furthermore, he was unable to walk due to spinal cord compression caused by metastatic epidural tumors. Therefore, the risk of preoperative DVT and PE may have been the highest. Fortunately, resuscitation was successful, and, thereafter, he underwent successful emergency pulmonary thrombectomy. He had no sequelae during follow-up. However, without preventive actions, lethal PE is an inevitable complication.

Vena cava filter placement is one method to prevent PE in patients with DVT [13]. Although the placement of a vena cava filter cannot fully prevent PE [14], the method may be useful for the prevention of PE after spinal surgery [15]. In addition, the benefit of rivaroxaban in the prevention and treatment of thrombosis in patients with AT III deficiency has recently been reported [16-19]. Oral anticoagulants increase the risk for epidural hematoma after spinal surgery but have a clear indication for the prevention of lethal PE in patients with congenital AT III deficiency.

Therefore, the prevention of thromboembolism, by maintenance of AT activity with replacement of AT products, by placement of a vena cava filter, and by the introduction of anticoagulants, should be prioritized before spinal surgery even in the presence of acute progressive paraplegia in patients with a metastatic spinal tumor associated with congenital AT III deficiency.

## Conclusions

In patients who are unable to walk due to a metastatic spinal tumor associated with congenital AT III deficiency, the prevention of thromboembolism should be prioritized before spinal surgery, even in the presence of acute progressive paraplegia.
